# Sequential extraction and organosolv pretreatment of halophytes: unlocking biomass recalcitrance for bio-based production

**DOI:** 10.1038/s41598-026-46584-w

**Published:** 2026-04-09

**Authors:** Maxwel Monção, Ahmed Al-Dubai, Aadila Cayenne, Hinrich Uellendahl, Ulrika Rova, Paul Christakopoulos, Leonidas Matsakas

**Affiliations:** 1 Biochemical Process Engineering, Division of Chemical Engineering, Department of Civil, Environmental and Natural Resources Engineering, Luleå University of Technology, 971 87 Luleå, Sweden; 2 Faculty of Mechanical and Process Engineering and Maritime Technologies, Flensburg University of Applied Sciences, Kanzleistr. 91-93, 24943 Flensburg, Germany

**Keywords:** *Salicornia*, Halophyte, Biorefinery, Organosolv fractionation, Anaerobic digestion, Biological techniques, Biotechnology, Environmental sciences, Plant sciences

## Abstract

The growing demand for sustainable bioresources has given prominence to lignocellulosic biomass as a possible alternative. Halophytes offer a specific advantage in this context, because they can grow on non-arable land with minimal freshwater requirements. To our knowledge, this is the first study to integrate organosolv fractionation to either Soxhlet extraction (SLE) or subcritical water extraction, (SWE) at 140 °C, as pretreatment strategies, into a single sequential process applied to halophyte biomass allowing the recovery of bioactive molecules from halophyte biomass. The spent fibers underwent organosolv fractionation under varied process conditions (180–200 °C, 15–60 min, 40–60% v/v ethanol) and were subsequently evaluated for bioenergy production via anaerobic digestion. Organosolv fractionation generates separate lignin and hemicellulose streams, which can be further converted into value-added products. Either extraction methodology effectively facilitated subsequent biomass fractionation. SLE-pretreated fibers attained > 96.4% hemicellulose removal in 87.5% of the tested conditions and 78.1% delignification. SWE-pretreated fibers exhibited slightly lower hemicellulose removal at higher (60% v/v) ethanol content and 69.3% delignification. Crucially, SWE fibers demonstrated superior bioenergy potential, achieving a methane yield of 336 mL-CH_4_/gVS, and complete saccharification in 62.5% of tested conditions. Our findings highlight the potential of synergistic pretreatment strategies in an integrated biorefinery approach, which valorizes halophytes as a sustainable feedstock for bio-based applications.

## Introduction

The need to reduce our reliance on fossil fuels is driving research on alternatives with lower environmental impact. Lignocellulosic materials are a key element in the transition to a fossil-free society by providing essential building blocks for chemicals used in everyday products^1^. Plants contain a complex network of biological components, including structural elements and secondary metabolites, such as carbohydrates, lignin, proteins, lipids, and vitamins^2,3^. Their composition varies by species, location, age, and season^4–6^. To optimize the recovery of all the above molecules, efficient multi-step plant fractionation is required^7^.

The choice of feedstock is crucial for its biorefinery application, as it influences transformation and transportation costs, as well as the possibility to produce specific molecules^8,9^. To prevent competition with food production and the use of arable land, alternative plant sources must be considered. Halophytes offer a possible solution, as these salt-tolerant plants thrive where traditional forestry or agriculture crops cannot grow^10^. Halophytes of the *Salicornia* species are a widespread source of bioactive compounds with potential applications in cosmetics^11,12^, feed formulation^13,14^, antioxidants, anti-inflammatories^15^, and antimicrobials^16^. Previous studies, including by our group, have explored their potential in biorefinery applications via organosolv fractionation and anaerobic digestion^17–21^. *Salicornia ramosissima* is an annual plant, whose growth cycle is dictated by temperature, salinity, and rainfall. In the spring and early summer, the seeds sprout into new individuals that grow vegetatively as succulent plants, before reaching the reproductive stage in mid or late summer, when flowers and seeds appear. Finally, they enter a senescent stage in late summer or fall, whereby tissues start to dry out and become lignified once seed dispersal is over. Besides primary metabolites, such as carbohydrates, proteins, and chlorophyll; secondary metabolites produced during the vegetative stage include amino acids (e.g. proline), and osmoprotectants that safeguard the plants against salt stress^22,23^. During the reproductive stage, the plants produce flavonoids, polyphenols, saponins, lipids, and phenolic acids (e.g., ferulic acid), along with the accumulation of lipids^23,24^. The senescent stage is accompanied by deposition of lignin, increased production of phenolic compounds, tannins, and fatty acids in seeds, as well as increased mineralization due to a diminishing water content^23^. In the present study, we assessed the potential of using lignified fibers from late-harvest *Salicornia* (i.e., after seed production, used as a source of both food and energy^25,26^.

Salicornia plants have been widely recognized for their potential as saline-tolerant crops, with successful cultivation demonstrated both in greenhouse systems and in open-field saline soils^27,28^, including aquaponics systems^29,30^. Several pilot and commercial cultivation initiatives, particularly in Europe, highlight that Salicornia is no longer an exploratory specimen but a crop under active agronomic development^28,31^. This growing interest stems from its suitability for marginal lands, its seed oil potential, and its emerging role as a green alternative to fossil-derived resources. In this context, our study contributes to the ongoing development of Salicornia as a viable crop by demonstrating its added value when integrated into a multistep biorefinery process.

Aiming for a holistic recovery and utilization of compounds from *Salicornia* biomass, we applied a sequence of optimized processing steps. Soxhlet extraction (SLE) and subcritical water extraction (SWE) enable the recovery of a broad range of polar and moderately polar compounds, including phenolics, glycosides, alkaloids, proteins, and organic acids^32–34^. The residual pulp can then serve as feedstock for biomass biorefineries.

Organosolv fractionation is an efficient method for the separation of cellulose, lignin, and hemicelluloses present in the plant cell wall^35,36^. The process is typically performed at 120–200 °C for a duration of 30–90 min, using an aqueous mixture of organic solvents, such as ethanol, methanol, and acetone^36^. Process optimization is key to obtaining highly pure fractions suitable for downstream applications in a biorefinery approach. Lignin can serve as a source of platform chemicals, and can be further developed into adhesives, coatings for controlled-release fertilizers, and drug delivery systems^37–39^; hemicelluloses can be utilized as a source of C5 and C6 sugars, prebiotics, emulsifiers, and fermentation feedstock^1^; and cellulose can serve as a valuable substrate for fermentations, sustainable packaging, and renewable energy production via anaerobic digestion^19,40,41^. Pilot scale of organosolv processes have been demonstrated^36^, indicating that such process has potential to be used in the fractionation of biomass with industrially validated approaches for producing high-purity lignin and hemicellulose streams. Among the different solvents, ethanol organosolv systems present low-toxicity, low-viscosity, and is fully compatible with conventional separation and solvent-recovery operations^40–43^.

The specific aim of this study was to compare the effect of two different extraction methods, SLE and SWE, each individually followed by organosolv fractionation of the spent fibers and subsequent application of the pretreated cellulose-rich pulp in anaerobic digestion for the production of bio-methane. This work introduces a previously unreported process sequence for halophyte valorization in order to enable a holistic component fractionation and recovery, and downstream bioenergy generation. The choice of late-harvest Salicornia fibers can potentially allow multipurpose valorization where succulent shoots can potentially be applied to food, and the senescent biomass is retained long enough to ensure seed production, whose oil is valuable for both food and bioenergy as previously reported.

## Materials and methods

### Feedstock

*S. ramosissima* was grown in Portugal in sand soil and irrigated with a mixture of fresh water and brackish water aquaculture effluent. Woody *S. ramosissima* was harvested around 8 months after lignification and seed production^11^. The composition of untreated fibers (in % w/w, dry basis) was 24.92% cellulose, 21.02% hemicelluloses, 20.70% lignin, and 25.50% extractives. Spent fibers were obtained by either SLE or SWE. SLE was performed on 2 kg of milled particles > 2 mm in size, which were treated for 8 h with 25 L of demineralized water. SLE was carried out at a pilot-scale Soxhlet plant located at Aalborg University in Esbjerg, Denmark. The composition of SLE-pretreated fibers of *S. ramosissima* (in % w/w, dry basis) was 25.80% cellulose, 27.98% hemicelluloses, 23.81% lignin, 17.66% extractives, and 0.87% ashes (total ashes in the extractives were 8.0%). Extraction by subcritical water (SWE) was performed at Celabor in Belgium. For the subcritical water extraction, 2 kg of shredded fibers were steeped twice with 35 L of tap water at room temperature. After filtration and drying, the fibers underwent two cycles of 30 min at 140 °C. The composition of SWE-pretreated fibers of *S. ramosissima* (in % w/w, dry basis) was 28.55% cellulose, 28.26% hemicelluloses, 29.86% lignin, 9.12% extractives, and 1.22% ashes (total ashes in the extractives were 2.8%).

### Organosolv fractionation

**Fig. 1:**
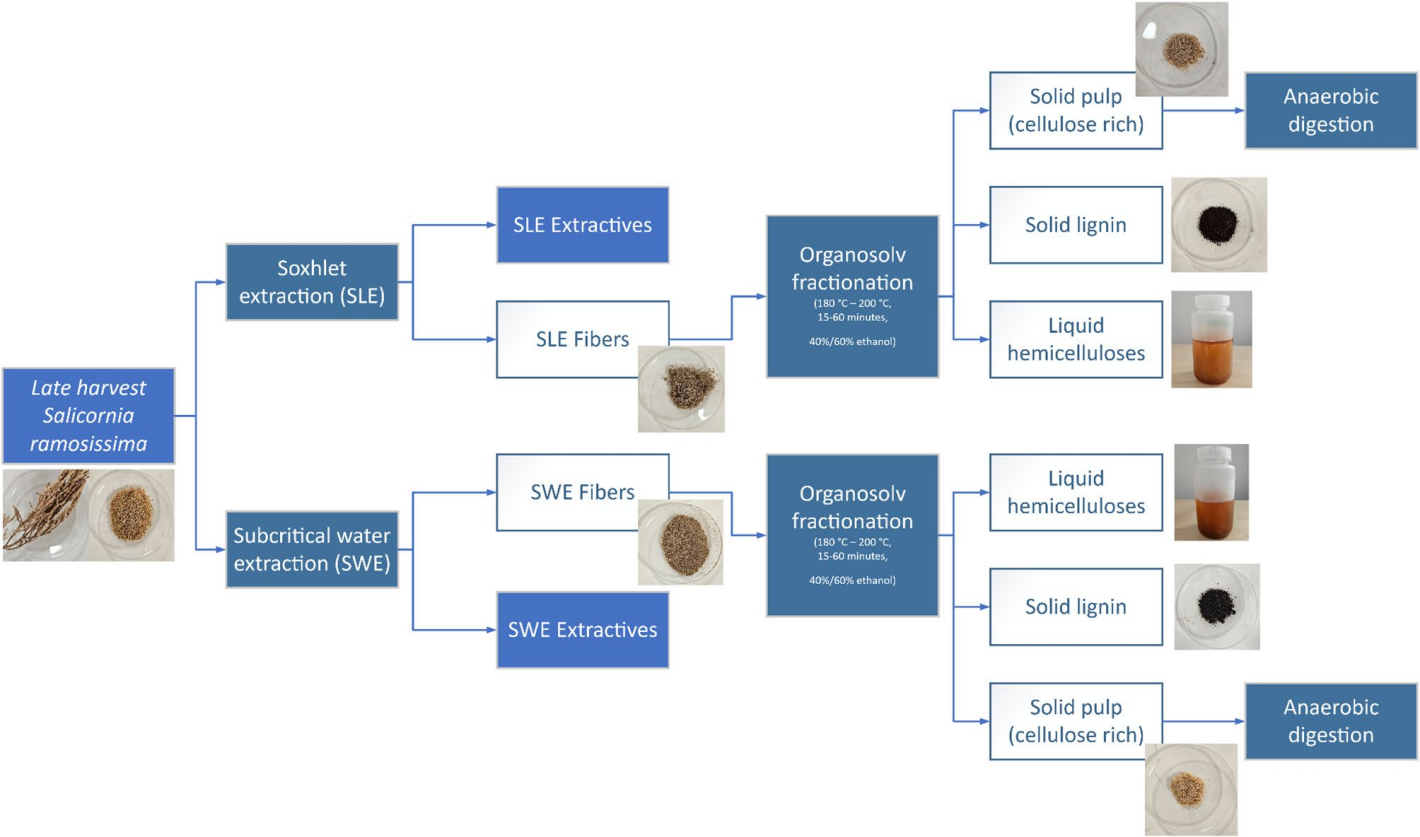
Overview of the processing of late harvest fibers from *S. ramosissima* through pretreatment, fractionation, and biomethane production.

Organosolv fractionation was performed in an air-heated organosolv reactor (Haato, Vantaa, Finland) loaded with six 2.5-L stainless steel batch reactors with rotational mixing The biomass load was 90 g (dry basis) and the solvent ratio was 1:10 w/v. Details about temperature, reaction time, and solvent composition are reported in Table 1. The solutions were heated to an average ratio of 1.5 °C/min. Once the residence time was over, the reactors were cooled down to 40 °C prior to vacuum filtration performed with filter paper of pore size 8–12 μm (Alhstrom Munksjö, Helsinki, Finland) so that solid pretreated fibers (pulp) could be separated from the liquor. The pulp was air-dried, moisture content was evaluated gravimetrically, and samples were stored in plastic bottles at room temperature. Instead, the liquor was processed in a rotary-evaporator (Heidolph, Schwabach, Germany) to collect the ethanol, followed by centrifugation at 12,000 × *g* for 10 min at 4 °C (5804R; Eppendorf, Hamburg, Germany) to separate lignin (i.e., solid fraction) from hemicellulose (i.e., liquid fraction). The lignin was freeze-dried (Telstar, Terrassa, Spain), while the liquid was bottled in plastic containers and stored at -20 °C. An overview of pretreatments and organosolv fractionation is shown in Fig. 1.

**Table 1:** Pretreatment and organosolv fractionation conditions applied to late harvest *S. ramosissima* fibers.

CODE	Pretreatment	Temperature (°C)	Time (min)	Ethanol content (% v/v)
SLE-0A4	Soxhlet	200	15	40
SLE-0A6	Soxhlet	200	15	60
SLE-0B4	Soxhlet	200	30	40
SLE-0B6	Soxhlet	200	30	60
SLE-1B4	Soxhlet	180	30	40
SLE-1B6	Soxhlet	180	30	60
SLE-1D4	Soxhlet	180	60	40
SLE-1D6	Soxhlet	180	60	60
SWE-0A4	Subcritical water	200	15	40
SWE-0A6	Subcritical water	200	15	60
SWE-0B4	Subcritical water	200	30	40
SWE-0B6	Subcritical water	200	30	60
SWE-1B4	Subcritical water	180	30	40
SWE-1B6	Subcritical water	180	30	60
SWE-1D4	Subcritical water	180	60	40
SWE-1D6	Subcritical water	180	60	60

### Analysis

Cellulose and lignin fractions were characterized using the NREL/TP-510-42618 protocol^44^, while the liquid fraction was analyzed following the NREL/TP-510-42623 protocol^45^. The analysis was performed in duplicates. Sugars were quantified by high-performance anion-exchange chromatography (HPAEC) (Thermo Fisher Scientific, Waltham, MA, USA) with a CarboPac PA-20 column (3 × 150 mm; Dionex™, Thermo Fisher Scientific) and a pulsed amperometric detector equipped with a gold electrode. The samples were eluted at 0.4 mL/min with a combination of buffer A (deionized water), buffer B (200 mM NaOH), and buffer C (100 mM Na acetate in 100 mM NaOH). Elution was as follows: 0–18 min at 98.8% A and 1.2% B; 18–20 min at a gradient of 1.2%–50% B; 20–30 min at 50% A and 50% B; 30.1–46 min at 100% C; 46.1–50 min at 100% B; and 50.1–60 min at 98.8% A and 1.2% B. The recovery of different components in the solid pulps were calculated according to Eq. 1:

1 $$Recovery = 100 \times \left( {\frac{{ \mathrm{\%} C_{{PRETREATED}} \times pretreated\;solids_{{MASS}} }}{{ \mathrm{\%} C_{{UNTREATED}} \times initial\;biomass_{{MASS}} }}} \right)$$

where C indicates the component (i.e.: lignin, cellulose, or hemicellulose) in untreated biomass and pulp fractions.

Sugar degradation compounds in the liquid fractions were detected by high-performance liquid chromatography (HPLC; Agilent Technologies, Santa Clara, CA, USA) using an Aminex HPX-87 H column (Bio-Rad, Hercules, CA, USA) and a UV detector set to 205 nm for formic acid, 227 nm for acetic acid, or 280 nm for levulinic acid, hydroxymethylfurfural, and furfural. The degradation compounds’ yields were calculated according to Eq. 2.

2 $$Degradation\;compound = \frac{{concentration\,\left( {mg \times mL^{{ - 1}} } \right) \times liquid\;fraction\;volume\,\left( {mL} \right)}}{{Mass\;of\;initial\;untreated\;biomass\,\left( g \right)}}$$

The HPLC was operated at 65 °C with a flow of 0.6 mL/min and mobile phase of 5 mM H_2_SO_4_. Extractives were quantified following the NREL/TP-510-42619 protocol^46^ using a Soxhlet extractor with a sequence of water, ethanol, and a 2:1 chloroform: methanol mixture. Water extractives were freeze-dried, while ethanol and chloroform: methanol were first evaporated, and the water-insoluble extractives were dried. The fractions were then quantified and stored at room temperature. Total solids (TS), volatile solids (VS), and ash content were measured following the established protocols DIN 12,879 and DIN 12,880^47,48^. TS were evaluated by drying samples at 105 °C until constant weight was attained (approximately 24 h); whereas VS were determined by incinerating TS at 550 °C for 3 h in a muffle furnace (M104; Thermo Fisher Scientific). Ash content was measured by weighing the inorganic residue left after incineration. The mineral content, in terms of sodium (Na) of the biomass material was analyzed by inductively coupled plasma optical emission spectroscopy (iCAP6000; Thermo Fisher Scientific) at Leibniz University, Hannover, Germany, in accordance with standard methods highlighted by Cayenne et al.^21^.

### Size-exclusion chromatography of cellulose

Holocellulose, which comprises cellulose and hemicellulose, was prepared as described by Meng et al.^49^. Specifically, about 0.6 g of sample was delignified with 6 mL of 5% w/w peracetic acid for 24 h at 25 °C in a water bath. The samples were then washed with deionized water and air-dried overnight. α-Cellulose was isolated from holocellulose by solubilization in 6 mL of 17.5% w/w NaOH for 2 h, followed by 8.75% NaOH for an additional 2 h. The cellulose was obtained by filtration and washing with 1% acetic acid and an excess of deionized water until the pH of the filtrate reached close to 7 and then air-dried in a fume hood. Cellulose (15 mg) was derivatized using 4 mL of anhydrous pyridine and 0.5 mL of phenyl isocyanate over 48 h at 70 °C. The reaction was quenched by adding 1 mL of anhydrous methanol. A methanol and water mixture (7:3, v/v) was added dropwise (10 mL) to promote precipitation of the cellulose derivative. The solids were collected by filtration and washed with 50 mL of the methanol-water-mixture, followed by 50 mL of water. The cellulose derivative was then dried overnight. Prior to gel permeation chromatography (GPC), the cellulose derivative was dissolved overnight in tetrahydrofuran (1 mg/mL), after which the solution was filtered through a 0.45-µm PTFE syringe filter and placed in a 2-mL auto-sampler vial. The molecular weight distribution was analyzed by HPLC (PerkinElmer, Waltham, MA, USA) at 40 °C with tetrahydrofuran as mobile phase and a flow rate of 0.6 mL/min. The system was equipped with a series of Styragel^®^ HR 4E and HR 4 columns (Waters, Milford, MA, USA) and a UV detector set to 280 nm. Micro-crystalline cellulose (Avicel^®^; Sigma- Aldrich, St. Louis, MO, USA) was used as reference after the derivatization step; the number average molecular weight (M_N_) was 1700 g/mol and the weight average molecular weight (M_W_) was 13,400 g/mol.

### Size-exclusion chromatography of lignins

The molecular weight of the lignin fraction was determined by GPC. First, acetobromination was performed using 5 mg of powdered lignin, 0.9 mL of glacial acetic acid, and 0.1 mL of acetyl bromide. The mixture was stirred at room temperature in amber vials for 2 h. Subsequently, acetic acid and acetyl bromide were evaporated in a rotary-evaporator and washed twice with 1 mL of tetrahydrofuran. The dried material was dissolved in tetrahydrofuran and filtered through 0.22-µm hydrophobic filters into HPLC vials. Samples were analyzed by HPLC (Agilent Technologies, Santa Clara, CA, USA) at 40 °C with tetrahydrofuran as mobile phase and a flow rate of 0.6 mL/min. The system was equipped with a Styragel^®^ HR 4E column (Waters, Milford, MA, USA) and UV detector set to 280 nm. Polystyrene standards of 474–100,000 Da (Sigma-Aldrich, St. Louis, MO, USA) were used to prepare the calibration curve.

### Pulp saccharification

The cellulose saccharification potential of pretreated pulp was assessed with the commercial cellulase enzyme solution Cellic^®^ CTec2 (Novozymes A/S, Bagsværd, Denmark). Incubation was carried out in duplicates at 50 °C and 900 rpm for 72 h in 2-mL microcentrifuge vials containing 3% w/w pulp in 50 mm citrate buffer (pH 5) with enzyme load of 20 FPU/g_solids_. Every 24 h, a set of vials was collected from the thermomixer, placed in a water bath at 95 °C for 5 min to deactivate the enzymes and then centrifuged at 10,000 × *g* for 10 min at room temperature. The supernatant was filtered using a 0.22-µm filter (Sartorius, Göttingen, Germany) and glucose was quantified by HPAEC as described previously^21^.

### Batch biomethane potential (BMP) tests

Batch assays to evaluate BMP were performed in triplicate using 0.5-L bottles in the Automated Methane Potential Test System II (Bioprocess Control, Lund, Sweden). The assays maintained mesophilic temperatures (37 ± 2 °C) with a substrate-to-inoculum ratio of 0.5, following the VDI 4630 standard^50^ as detailed by Cayenne et al. (2024)^19^. The inoculum used in the batch experiments was sourced from an anaerobic digester at a wastewater treatment plant in Flensburg, Germany, which operates under mesophilic conditions. TS and VS in the inoculum were 2.0% w/w and 59.7% w/w, respectively. Each batch vial was stirred mechanically for 10 min every hour. Control batches containing solely the inoculum were set up to assess methane yield from sludge and from the added biomass substrate. The initial pH of 7.6 ± 0.4 remained stable throughout the 34-day digestion process.

### Theoretical methane potential

The theoretical biomethane potential (TBMP_OFC_) of a substrate can be calculated using its organic fraction composition (OFC) via Buswell’s formula (Eq. 3)^51,52^:

3 $${\mathrm{TBMP}}_{{{\mathrm{OFC}}}} \left( {{\mathrm{mL}} - {\mathrm{CH}}_{{\mathrm{4}}} /{\mathrm{gVS}}} \right) = 415 \times \% Carbohydrates + 496 \times \% Proteins + 1014 \times \% Lipids$$

The TBMP_OFC_ in this study was calculated based solely on carbohydrate content, as lipid and protein contents were assumed to be negligible. This assumption stems from the fact that *Salicornia* biomass was harvested at a mature stage rather than at a vegetative stage, which means a strongly lignified plant. A previous study demonstrated that organosolv fractionation of de-juiced green fibers reduced crude protein (CP) content in *Salicornia dolichostachya* from 8 to 1–2% (TS, w/w) and in *S. ramosissima* from 13 to 7–9% (TS, w/w), respectively. This indicated that proteins were solubilized into the process liquor during organosolv fractionation, and the residual fibers contained primarily carbohydrates^19^. Given this evidence, it is reasonable to infer that the lignified fibers in this study would have an even lower protein content after cascade processing.

### Anaerobic biodegradability

The extent of biodegradability (BD) of a substrate was calculated using Eq. 4, which compared the experimental biomethane yield (BMP_EXP_) to its TBMP_OFC_ derived from organic composition^52^.

4 $${\mathrm{BD}}\,\left( \% \right) = \frac{{BMP_{{EXP}} }}{{TBMP_{{OFC}} }} \times 100$$

## Results and discussion

### Organosolv fractionation

#### Pulp fraction

Pulp recovery and composition after organosolv fractionation is shown in Table 2. Overall, organosolv achieved high fiber solubilization, with recovery of insoluble solid fibers reaching 51% following SLE and 64% following SWE. Under identical conditions, fibers recovered from SLE samples amounted to only 75.7%–86.9% of those recovered from SWE samples, indicating that SLE fibers were more sensitive to fractionation.

**Table 2:** Composition of the pulp in terms of cellulose, hemicelluloses, lignin, and ashes (in % w/w) of organosolv fractionated fibers.

Code	Solid fibers recovery (%)	Cellulose (%Recovery)	Hemicellulose (%Recovery)	Lignin (%Recovery)	Ash (%Recovery)	Cellulose M_*N*_	Cellulose M_W_	Dispersity index (M_W_/M_*N*_)
SLE-0A4	44.54	57.38 (81.57)	1.21 (1.59)	29.24 (45.04)	1.9 (10.58)	3,400	29,100	8.56
SLE-0A6	42.11	63.39 (85.19)	1.72 (2.13)	17.48 (25.45)	2.9 (15.26)	3,400	27,700	8.15
SLE-0B4	42.24	60.33 (81.33)	1.20 (1.49)	27.51 (40.19)	2.0 (10.56)	3,300	28,100	8.52
SLE-0B6	40.60	67.15 (87.00)	1.67 (1.99)	15.61 (21.91)	3.2 (16.24)	3,200	26,700	8.34
SLE-1B4	50.91	53.91 (87.60)	6.53 (9.78)	30.36 (53.45)	1.9 (12.09)	3,400	27,700	8.15
SLE-1B6	50.98	57.01 (92.76)	2.39 (3.58)	17.21 (30.35)	2.8 (17.84)	3,400	26,900	7.91
SLE-1D4	45.14	62.65 (90.27)	1.52 (2.02)	30.44 (47.52)	2.0 (11.29)	3,300	27,100	8.21
SLE-1D6	43.77	67.94 (94.91)	1.97 (2.53)	19.54 (29.58)	3.0 (16.41)	3,300	26,900	8.15
SWE-0A4	55.04	49.98 (87.55)	1.40 (2.48)	29.78 (49.88)	3.0 (58.97)	3,200	26,500	8.28
SWE-0A6	48.44	58.92 (90.86)	13.88 (21.63)	20.85 (30.74)	2.0 (34.60)	3,200	25,500	7.97
SWE-0B4	52.89	52.36 (88.15)	1.06 (1.81)	33.12 (53.32)	2.5 (47.23)	3,100	25,700	8.29
SWE-0B6	53.63	52.61 (89.82)	17.35 (29.93)	19.64 (32.06)	3.0 (57.46)	3,200	25,700	8.03
SWE-1B4	61.50	49.24 (96.40)	13.34 (26.38)	22.83 (42.73)	2.5 (54.91)	3,100	25,400	8.19
SWE-1B6	64.36	46.31 (94.87)	23.84 (49.34)	23.67 (46.36)	2.4 (55.16)	3,100	25,700	8.29
SWE-1D4	56.44	52.47 (94.27)	6.15 (11.17)	31.26 (53.71)	2.0 (40.32)	3,200	25,900	8.09
SWE-1D6	57.37	51.08 (93.29)	16.17 (29.84)	22.24 (38.84)	2.4 (49.17)	3,000	24,800	8.27

M_N_: Number average molecular weight; M_W_: weight average molecular weight. SLE, Soxhlet-extracted fibers; SWE, subcritical water-extracted fibers; 0, 200 °C; 1, 180 °C; A, 15 min; B, 30 min; D, 60 min; 4, 40% v/v ethanol; 6, 60% v/v ethanol.

Biomass pretreated with SLE prior to organosolv fractionation yielded pulp with a higher cellulose content compared to biomass pretreated with SWE. However, because the general amount of solids from organosolv fractionation was lower after SLE, the recovery of cellulose was actually higher after SWE. Cellulose content in the fibers increased by 2.09–2.63-fold upon SLE and by 1.62–2.06-fold upon SWE compared to untreated fibers. With both feedstocks, more cellulose was recovered with fractionations performed at 180 °C (88%-95% for SLE and 94–96% for SWE) than at 200 °C or when 40% v/v ethanol was employed at 200 °C.

Hemicellulose content in the pulp was below 2.4% w/w in all but the mildest fractionation of SLE fibers (SLE-1B4). In contrast, for SWE fibers, it reached 6.15% w/w to 23.84% w/w in six out of eight cases. We observed a strong correlation between solvent composition and the amount of hemicelluloses remaining in the pulp. Fractionations using 60% v/v ethanol yielded more hemicelluloses in the pulp; the only exception was the mildest SLE fractionation (180 °C for 30 min). Among SLE fibers, hemicellulose content varied slightly based on solvent composition; whereas for SWE fibers, a 2–16-fold increase was observed when using 60% v/v as opposed to 40% v/v ethanol. Therefore, 60% v/v ethanol is not effective for hemicellulose removal in SWE fibers.

The concentration of Klason lignin in the pulp correlated with ethanol content during organosolv, with 60% v/v ethanol yielding less lignin (i.e., higher delignification) for both pretreatments. The lowest recovery of lignin was obtained from fractionations performed at 200 °C with 60% v/v ethanol: 25.5% and 21.9% for SLE fibers treated for 15 min and 30 min, respectively. Kubota et al.^53^ studied the effect of direct organosolv versus a sequential route comprising SWE at 120 °C to remove extractives, followed by SWE at 180 °C to remove hemicelluloses, and finally organosolv for the delignification of *Miscanthus x giganteus*. The authors observed lower delignification of fibers pre-extracted by SWE^53^.

The trends observed, such as increased delignification at 200 °C and increased hemicellulose recovery with higher ethanol concentration (60%), are consistent with classical organosolv behavior for wood and agricultural residues as well as our prior work with *S. dolichostachya*^17^.

Ashes in the pretreated solids amounted to 1.9%–3.2% w/w, which could be explained by Na accounting for 60.5 mg/g of the untreated biomass^21^. Ash content was reduced by 64% and 87% following SLE and SWE pretreatments, respectively, and the resulting fibers contained 32 mg/g and 3 mg/g Na. These values represented a drop of 47% and 95% in Na content compared to untreated fibers. Organosolv fractionation led to a 60%–76% reduction in ash content for SLE fibers and a 10%–29% reduction for SWE fibers. Accordingly, Na decreased to 4–5 mg/g and 1–2 mg/g, respectively, upon SLE or SWE and subsequent organosolv fractionation.

Cellulose M_W_ values fell within a close range of 24,800–29,100 g/mol, while M_N_ values varied only between 3,000 and 3,400 g/mol, suggesting relatively consistent polymer size distributions across samples. The dispersity index (DI) of cellulose fibers ranged from 7.9 to 8.6, indicating a relatively wide distribution of chain lengths and heterogeneity of the cellulose fractions. Interestingly, the DI remained consistent across all organosolv conditions.

#### Liquid hemicellulose fraction

**Fig. 2:**
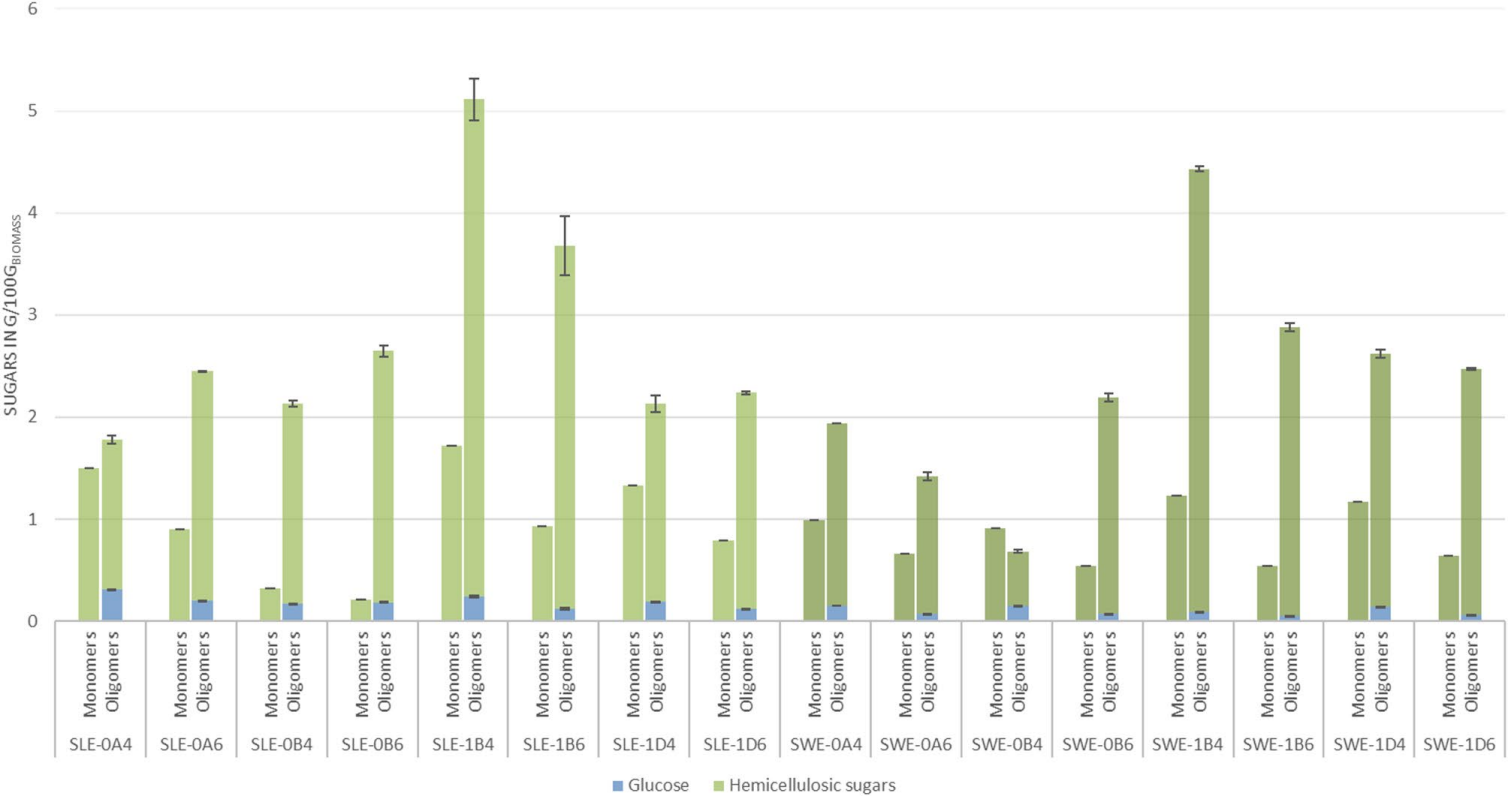
Total sugars (in g per 100 g of untreated biomass) expressed as monomeric sugars present in hemicellulose stream. Codes: SLE, Soxhlet-extracted fibers; SWE, subcritical water-extracted fibers; 0, 200 °C; 1, 180 °C; A, 15 min; B, 30 min; D, 60 min; 4, 40% v/v ethanol; 6, 60% v/v ethanol. Error bars represent standard deviation.

After organosolv fractionation, the liquid stream contained solubilized hemicelluloses in the form of monomeric sugars and soluble oligomers (Fig. 2). Fractionation of SLE and SWE fibers produced similar ratios of sugars irrespective of organosolv treatment. The mildest fractionation (180 °C for 30 min with 40% v/v ethanol) yielded the highest recoveries of hemicellulose sugars for both SLE (total 6.8 g/100g_BIOMASS_) and SWE (total 5.7 g/100g_BIOMASS_). With exception to the organosolv operations at 180 °C for 60 min, SLE fibers yielded higher hemicellulose sugars compared to SWE.

Harsher organosolv conditions, such as higher temperature and longer reaction time, promote further hydrolysis of hemicelluloses into monomeric sugars^54^. For SLE fibers, the lowest proportion of oligomers (54%) to total recovered hemicelluloses was obtained upon fractionation at 200 °C for 15 min with 40% v/v ethanol. Similarly, the lowest ratio of oligomeric sugars from SWE fibers (43%) was also achieved with 200 °C with 40% v/v ethanol, but for a duration of 30 min. In all the fractionations for both feedstocks, a higher ratio of oligomeric sugars was obtained from the organosolv fractionations performed with 60% v/v ethanol when compared to operations performed at same temperature and residence time with solvent composition of 40% v/v ethanol. In our previous study on early-harvest *S. ramosissima*, the ratio of oligomers was also higher with 60% v/v ethanol in pretreatments at 200 °C whereas solvent composition did not have a significant impact for pretreatments at 160 °C and 180 °C^18^. In the current study, the highest ratio of oligomers was found in SLE-0B6 (92.5%). Individually, the highest recovery of sugars in the liquid fraction was from SLE-1B4 with 1.72 and 5.11 g/100g_BIOMASS_ monomers and oligomers, respectively.

Harsh conditions during the fractionation can lead to extended degradation of monomeric sugars. Sugar degradation occurs first by dehydration of hexoses into 5-Hydroxymethylfurfural (HMF) and pentoses into furfural and posterior degradation to form levulinic acid and formic acid while cleavage of acetyl groups leads to formation of acetic acid^55^. Aiming for possible downstream fermentation of hemicellulose sugars, we quantified the sugar degradation compounds present in the recovered liquid hemicellulose fraction (Table 3). Under all tested conditions, the use of 40% v/v ethanol resulted in higher amounts of degradation compounds compared to 60% v/v ethanol. This can be attributed to the greater autoionization of water at lower ethanol concentrations, which leads to more H^+^ ions in solution and, consequently, more extensive sugar degradation^56^. Among SWE fibers, higher temperature during the organosolv fractionation yielded higher degradation compounds whereas the same trend was not observed in SLE fibers. With exception to SLE-0B4, formic acid was the major compound among the tested degradation compounds. Values for acetic and levulinic acid were negligible.

**Table 3:** Presence of sugar degradation products in the recovered liquid fraction after ethanol evaporation.

Code	Formic acid (mg/g_BIOMASS_)	Hydroxymethylfurfural (mg/g_BIOMASS_)	Furfural (mg/g_BIOMASS_)	Total (mg/g_BIOMASS_)
SLE-0A4	18.64 (0.55)	17.32 (0.20)	15.19 (0.02)	51.15
SLE-0A6	7.18 (0.94)	1.82 (0.02)	0.40 (0.00)	9.40
SLE-0B4	19.79 (1.18)	20.49 (0.52)	5.97 (0.22)	46.25
SLE-0B6	11.82 (0.29)	11.81 (0.38)	5.65 (0.09)	29.28
SLE-1B4	20.48 (1.79)	14.15 (0.49)	6.23 (0.18)	40.86
SLE-1B6	9.07 (0.27)	5.88 (0.44)	1.57 (0.07)	16.52
SLE-1D4	19.24 (0.61)	16.85 (0.57)	17.13 (1.36)	53.23
SLE-1D6	6.05 (0.07)	1.98 (0.36)	1.39 (0.16)	9.42
SWE-0A4	17.48 (0.29)	8.08 (0.42)	7.24 (0.06)	32.80
SWE-0A6	7.36 (0.43)	3.94 (0.25)	4.43 (0.38)	15.72
SWE-0B4	24.03 (1.44)	16.99 (0.44)	10.03 (0.24)	51.05
SWE-0B6	8.61 (0.35)	3.78 (0.17)	0.86 (0.07)	13.26
SWE-1B4	13.87 (0.72)	6.32 (0.53)	2.86 (0.06)	23.04
SWE-1B6	6.32 (0.27)	2.16 (0.25)	0.87 (0.06)	9.35
SWE-1D4	9.56 (0.22)	7.91 (0.08)	4.76 (0.18)	22.24
SWE-1D6	6.63 (0.25)	3.32 (0.02)	1.27 (0.08)	11.22

Values are presented as mg/g of untreated biomass. SLE, Soxhlet-extracted fibers; SWE, subcritical water-extracted fibers; 0, 200 °C; 1, 180 °C; A, 15 min; B, 30 min; D, 60 min; 4, 40% v/v ethanol; 6, 60% v/v ethanol. Values in braquets represent standard deviation. Acetic acid and levulinic acid were not detected in any sample.

#### Lignin fraction

The lignin fraction was analyzed for the presence of cellulose, hemicelluloses, lignin, and ashes as well as size exclusion chromatography (Table 4). For downstream application, the lignin fraction should have very few impurities, such as sugars and ashes.

**Table 4:** Composition of lignin fractions obtained from organosolv fractionation of SLE and SWE fibers.

Code	Glucose (% w/w)	Hemicelluloses (% w/w)	Klason Lignin (% w/w)	Ashes (% w/w)	M_*N*_ (g/mol)	M_W_ (g/mol)	Dispersity index (M_W_/M_*N*_)
SLE-0A4	0.11 (0.01)	1.13 (0.03)	89.58 (4.14)	0.73 (0.04)	1,000	3,000	3.00
SLE-0A6	0.00 (0.00)	2.46 (0.03)	84.06 (0.26)	0.81 (0.06)	900	2,900	3.22
SLE-0B4	0.05 (0.00)	1.37 (0.06)	89.19 (0.15)	1.32 (0.02)	800	2,500	3.13
SLE-0B6	0.00 (0.00)	2.51 (0.06)	90.64 (0.10)	0.50 (0.01)	800	2,600	3.25
SLE-1B4	0.05 (0.00)	3.59 (0.07)	84.36 (0.13)	0.43 (0.09)	1,200	5,200	4.33
SLE-1B6	0.08 (0.00)	6.96 (0.29)	81.32 (1.14)	0.98 (0.01)	1,100	3,500	3.18
SLE-1D4	0.23 (0.01)	2.09 (0.13)	82.48 (0.17)	1.11 (0.03)	700	1,900	2.71
SLE-1D6	0.00 (0.00)	3.20 (0.10)	91.50 (0.23)	1.03 (0.02)	800	2,500	3.13
SWE-0A4	0.05 (0.00)	3.20 (0.02)	83.50 (0.95)	0.24 (0.02)	800	2,200	2.75
SWE-0A6	0.06 (0.01)	5.68 (0.63)	82.07 (0.72)	0.20 (0.02)	800	2,400	3.00
SWE-0B4	0.19 (0.03)	0.49 (0.00)	88.16 (0.32)	0.26 (0.05)	800	2,000	2.50
SWE-0B6	0.70 (0.02)	6.62 (0.18)	83.68 (0.10)	0.43 (0.03)	800	2,600	3.25
SWE-1B4	0.09 (0.00)	9.21 (0.20)	76.99 (1.37)	0.39 (0.04)	1,000	2,900	2.90
SWE-1B6	0.07 (0.01)	8.56 (0.37)	82.44 (2.14)	0.53 (0.02)	1,300	4,700	3.62
SWE-1D4	0.06 (0.00)	3.44 (0.08)	82.06 (1.32)	0.38 (0.08)	800	2,300	2.88
SWE-1D6	0.06 (0.00)	6.33 (0.62)	83.80 (0.13)	0.45 (0.02)	800	2,300	2.88

M_N_: Number average molecular weight; M_W_: weight average molecular weight. SLE, Soxhlet-extracted fibers; SWE, subcritical water-extracted fibers; 0, 200 °C; 1, 180 °C; A, 15 min; B, 30 min; D, 60 min; 4, 40% v/v ethanol; 6, 60% v/v ethanol. Values in braquets represent standard deviation.

Except for the fractionation performed at 200 °C for 30 min with 40% v/v ethanol, SLE fibers presented fewer contaminating sugars than SWE fibers under the same organosolv conditions. Conversely, fractionated lignin from SWE fibers had less ashes (< 0.5% w/w) compared to that from SLE fibers (up to 1.3%).

For both feedstocks, the highest amount of impurities (including sugars and ashes) was present in lignin fractionated at 180 °C and 30 min: 8.0% w/w for SLE lignin and 9.7% w/w for SWE lignin. At higher temperature and longer residence time, lignin contamination with ashes and sugars did not surpass 4.2% w/w (SLE fibers) and 7.8% w/w (SWE fibers). Except for SWE-1B6, 40% v/v ethanol resulted in fewer impurities from SWE fibers. Fractionation performed at 200 °C for 30 min with 40% v/v ethanol yielded lignin with the lowest amount of impurities (0.9% w/w). Generally, SWE lignins contained more sugar contaminants compared to SLE lignins generated under the same organosolv conditions. Ashes contamination, instead, was higher in SLE lignins, although the percentage was not higher than 1.3% w/w.

The mildest organosolv conditions (180 °C for 30 min) produced molecules with higher M_W_: up to 5,200 g/mol for SLE and 4,700 g/mol for SWE. These conditions led also to the highest dispersity of lignin. Longer treatments (200 °C for 30 min and 180 °C for 60 min) promoted overall smaller lignin molecules.

#### Mass distribution of the major biomass fractions

**Fig. 3:**
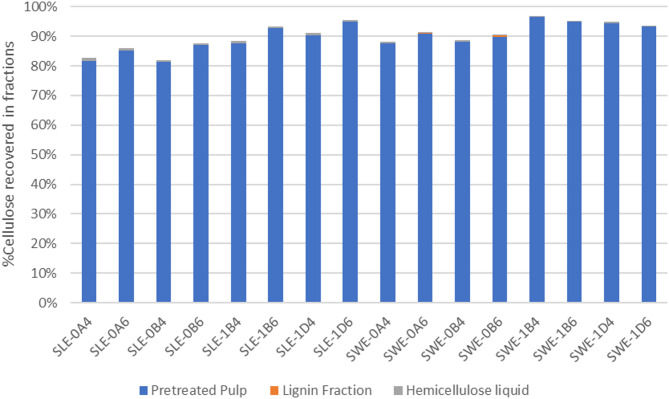
Cellulose mass balance across the three fractions produced from the organosolv process: pretreated pulp, lignin, and hemicellulose liquid. SLE, Soxhlet-extracted fibers; SWE, subcritical water-extracted fibers; 0, 200 °C; 1, 180 °C; A, 15 min; B, 30 min; D, 60 min; 4, 40% v/v ethanol; 6, 60% v/v ethanol.

Figure 3 illustrates the mass distribution of cellulose in the three fractions produced during organosolv treatment. No more than 1.1% of the cellulose initially present in the feedstock was found in the solid lignin fraction or in the remaining hemicellulose liquid. Major cellulose losses were observed upon fractionation at 200 °C with SLE fibers. For both feedstocks, cellulose recovery was higher following organosolv at 180 °C (> 88% for SLE and > 93% for SWE). The lower loss of cellulose in SWE fibers compared to SLE fibers under the same conditions, as well as the effect of other parameters on the recovery of cellulose will be discussed in the next section.

**Fig. 4:**
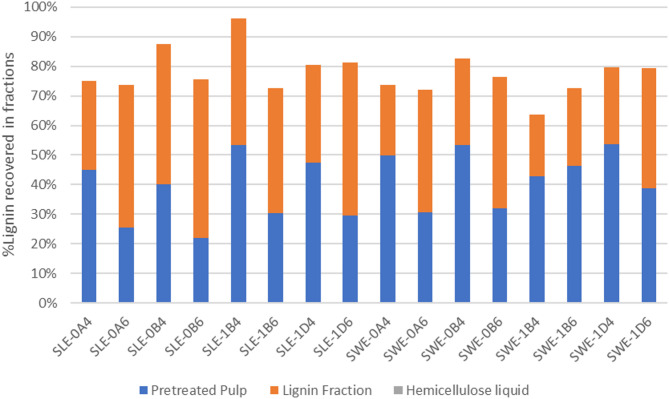
Lignin mass balance across the three fractions produced from the organosolv process: pretreated pulp, lignin, and hemicellulose liquid. SLE, Soxhlet-extracted fibers; SWE, subcritical water-extracted fibers; 0, 200 °C; 1, 180 °C; A, 15 min; B, 30 min; D, 60 min; 4, 40% v/v ethanol; 6, 60% v/v ethanol.

Figure 4 presents the mass distribution of lignin. Contrary to cellulose, lignin suffered bigger losses in SWE fibers than in SLE fibers under the same fractionation conditions. No significant amount of lignin was present in the hemicellulose liquid. SLE-0A4, SLE-1B4, and SLE-1D4 contained more lignin in the pulp than in the lignin fraction. Among SWE fibers, all fractionations performed with 40% v/v ethanol yielded more lignin (62.5%–65%) in the pulp than in the lignin fraction. The opposite was true for organosolv fractionations performed with 60% v/v ethanol (38.7%–60% lignin in the fraction).

**Fig. 5:**
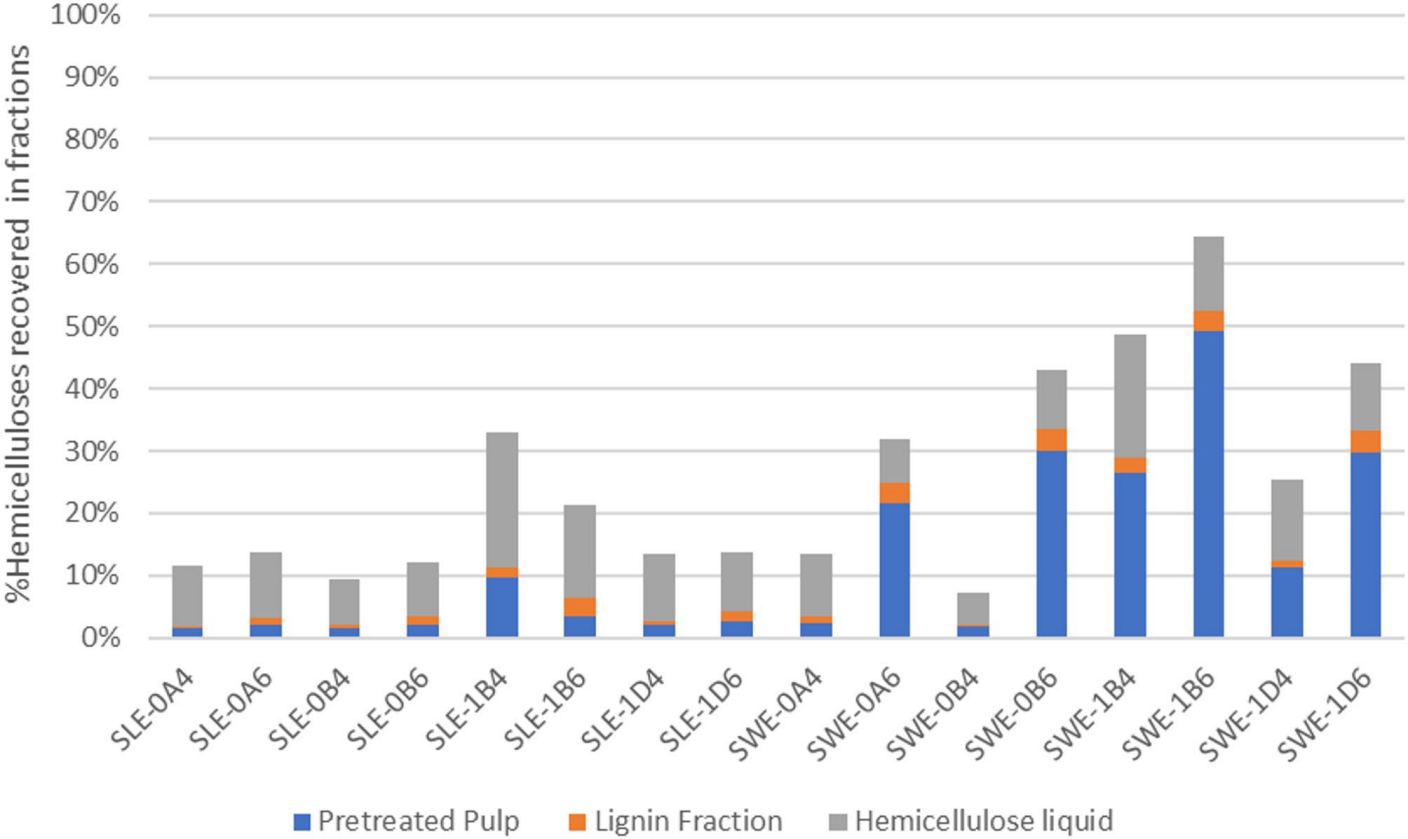
Hemicelluloses mass balance across the three fractions produced from the organosolv process: pretreated pulp, lignin, and hemicellulose liquid. SLE, Soxhlet-extracted fibers; SWE, subcritical water-extracted fibers; 0, 200 °C; 1, 180 °C; A, 15 min; B, 30 min; D, 60 min; 4, 40% v/v ethanol; 6, 60% v/v ethanol.

Figure 5 summarizes the distribution of hemicelluloses across the fractions produced following organosolv fractionation. Hemicelluloses suffered the most losses, particularly SLE fibers (> 67%), indicating greater sensitivity to organosolv fractionation. This can be traced to higher solubilization of the fibers. Smaller losses were recorded with the mildest treatments such as 180 °C for 30 min. In all cases, most hemicelluloses were recovered from the liquid fraction.

SWE fibers presented higher recovery of hemicelluloses across fractions. Major losses were observed following the harsher fractionations with 40% v/v ethanol (200 °C for 15 min or 30 min and 180 °C for 60 min). These three conditions achieved a similar distribution as with SLE fibers, with most hemicelluloses recovered from the liquid fraction (51%–74%). In contrast, all pretreatments performed with 60% v/v ethanol, as well as the mildest condition with 40% v/v ethanol (SWE-1B4), attained higher hemicellulose recovery from pretreated pulp (43%–76%), along with more hemicellulose-derived sugars (32%–65%). The degradation of hemicelluloses released during fractionation can explain this trend: when depolymerization is reduced, sugars become less exposed to secondary reactions and, therefore, are better protected from degradation.

#### Enzymatic hydrolysis of the pulp

**Fig. 6:**
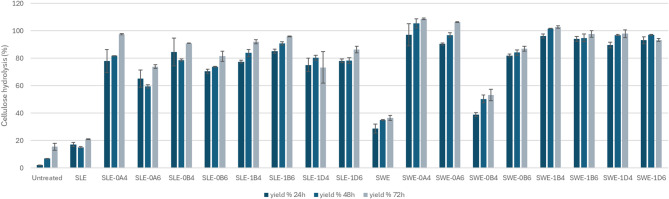
Glucose yield after saccharification for 24–72 h of organosolv-fractionated SLE and SWE fibers from *S. ramosissima*. SLE, Soxhlet-extracted fibers; SWE, subcritical water-extracted fibers; 0, 200 °C; 1, 180 °C; A, 15 min; B, 30 min; D, 60 min; 4, 40% v/v ethanol; 6, 60% v/v ethanol.

**Fig. 7:**
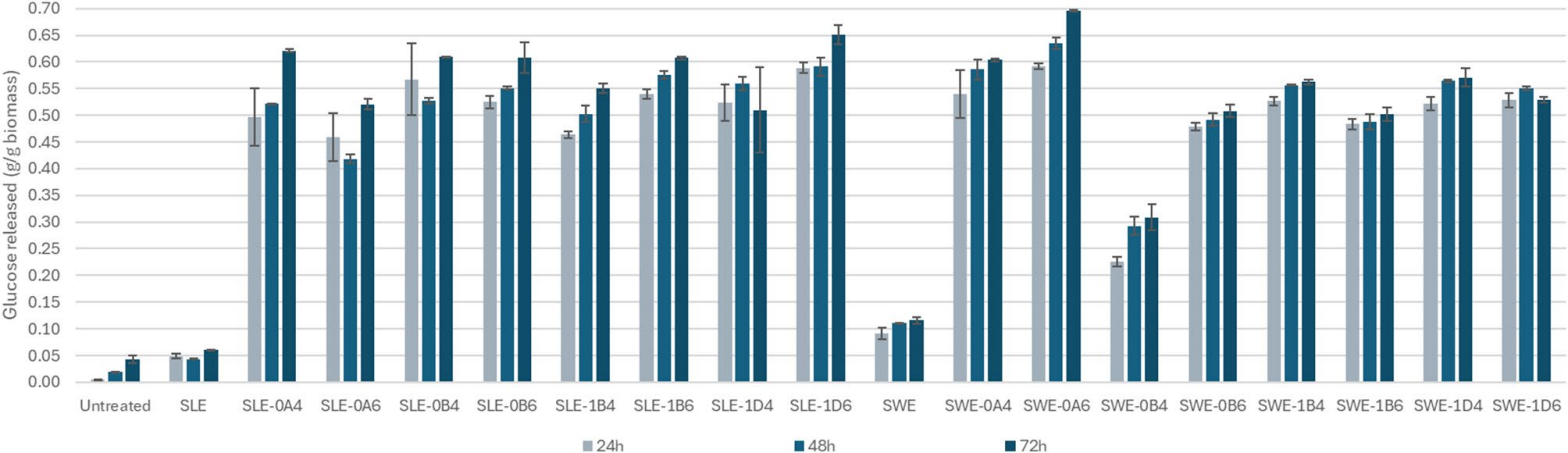
Glucose released during enzymatic saccharification for 24–72 h of organosolv-fractionated SLE and SWE fibers from *S. ramosissima.* Values are expressed in g/g of initial biomass. SLE, Soxhlet-extracted fibers; SWE, subcritical water-extracted fibers; 0, 200 °C; 1, 180 °C; A, 15 min; B, 30 min; D, 60 min; 4, 40% v/v ethanol; 6, 60% v/v ethanol.

Enzymatic hydrolysis of the pulp was conducted with the aim of determining the total carbohydrate content potentially available for subsequent bioconversion of organosolv-pretreated SLE and SWE fibers (Fig. 6). Overall, no strong correlations to fractionation conditions were observed. The lowest yield after incubation for 72 h was obtained with the SWE fraction that underwent organosolv pretreatment at 200 °C for 30 min with 40% v/v ethanol (0B4). Three out of eight SWE fibers achieved full saccharification (Fig. 6); whereas four SLE fibers attained > 90% theoretical saccharification and none attained full saccharification. Among the unfractionated fibers, SWE fibers also presented more susceptibility to the hydrolysis when compared to SLE (achieving 36.5% and 20.9%, respectively). Our previous study with the fresh fibers from *S. ramosissima*, none of the conditions fractionated at 180–200 °C achieved saccharification higher than 65% of the theoretical value, indicating that lignified fibers pretreated prior to fractionation present better saccharification yields^18^. To assess the glucose released in the medium during hydrolysis, we calculated the release of glucose in relation to the amount of biomass used prior to organosolv (Fig. 7).With exception to SWE-0B4, all the pretreated fibers released at least 0.502 g/g_BIOMASS_ with the highest release from SLE fibers of 0.651 g/g_BIOMASS_ (SLE-1D6) and among SWE fibers being 0.696 g/g_BIOMASS_ (SWE-0A6).

#### Methane potential and biodegradability of residual *Salicornia* fibers

Anaerobic digestion of halophytes faces two key challenges: high salt concentrations, which can impede microbial activity, and the presence of lignin in fibrous organic matter, which reduces both the rate and extent of hydrolysis during microbial degradation^19,21,57^. TS and VS of lignified *Salicornia* samples following the different extractions and organosolv treatments are presented in Table 5. The total lignocellulosic components of organosolv-treated lignified *Salicornia* fibers varied between 79% and 97% w/w of TS in SLE fibers, and between 81% and 94% w/w of TS in SWE fibers. This represents 79% to 97% of VS (based on TS) in SLE fibers and 83% to 96% of VS in SWE fibers after organosolv fractionation.

**Table 5:** TS, VS, and ash content of lignified *S. ramosissima* fibers after SLE and SWE followed by organosolv fractionation.

Substrate	TS	VS	VS
	w/w % DM	w/w % DM	w/w % TS
SLE Fibers	99.0 (0.4)	91.1 (0.5)	92.0 (0.2)
SLE-0A4	99.5 (0.0)	97.6 (0.2)	98.1 (0.1)
SLE-0A6	99.9 (0.0)	97.0 (0.1)	97.1 (0.1)
SLE-0B4	99.0 (0.2)	97.0 (0.3)	98.0 (0.1)
SLE-0B6	99.0 (0.2)	95.8 (0.4)	96.8 (0.3)
SLE-1B4	89.2 (0.2)	87.5 (0.1)	98.1 (0.1)
SLE-1B6	98.2 (0.1)	95.5 (0.2)	97.2 (0.2)
SLE-1D4	100 (0.0)	98.0 (0.1)	98.0 (0.1)
SLE-1D6	99.0 (0.4)	96.0 (0.5)	97.0 (0.1)
SWE Fibers	97.4 (0.2)	94.7 (0.1)	97.3 (0.1)
SWE-0A4	99.0 (0.1)	96.0 (0.0)	97.0 (0.1)
SWE-0A6	99.0 (0.1)	97.0 (0.1)	98.0 (0.1)
SWE-0B4	98.5 (0.0)	96.1 (0.3)	97.5 (0.2)
SWE-0B6	99.3 (0.1)	96.3 (0.3)	97.0 (0.4)
SWE-1B4	72.1 (0.4)	70.3 (0.7)	97.5 (0.4)
SWE-1B6	86.2 (0.1)	84.1 (0.5)	97.6 (0.6)
SWE-1D4	98.8 (0.1)	96.9 (0.1)	98.0 (0.1)
SWE-1D6	99.4 (0.0)	97.0 (0.1)	97.6 (0.1)

SLE, Soxhlet-extracted fibers; SWE, subcritical water-extracted fibers; 0, 200 °C; 1, 180 °C; A, 15 min; B, 30 min; D, 60 min; 4, 40% v/v ethanol; 6, 60% v/v ethanol.

**Fig. 8:**
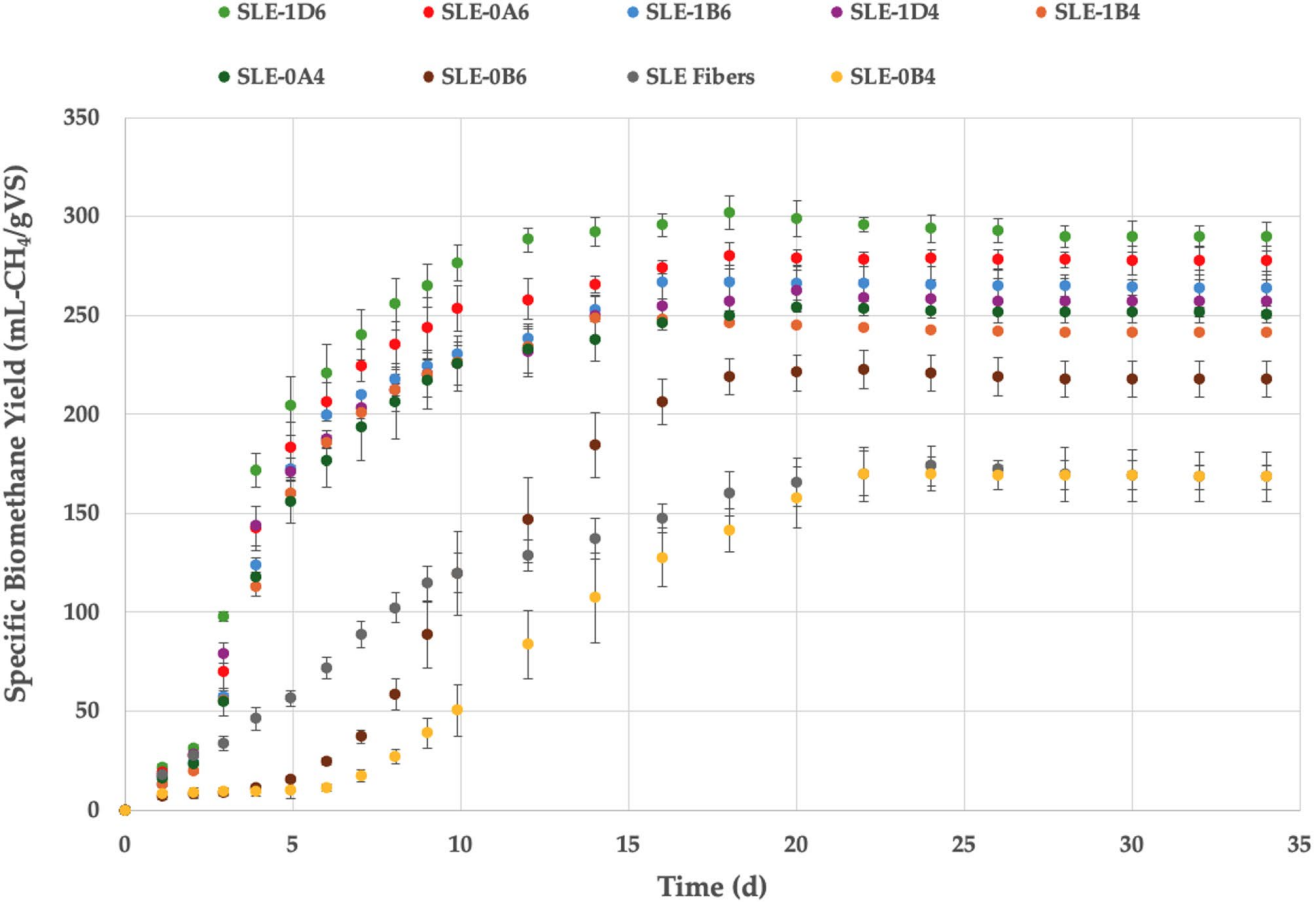
Specific biomethane yield in AD batch experiment of *S. ramosissima* fibers after SLE alone or SLE and subsequent organosolv fractionation. SLE, Soxhlet-extracted fibers; SWE, subcritical water-extracted fibers; 0, 200 °C; 1, 180 °C; A, 15 min; B, 30 min; D, 60 min; 4, 40% v/v ethanol; 6, 60% v/v ethanol.

**Fig. 9:**
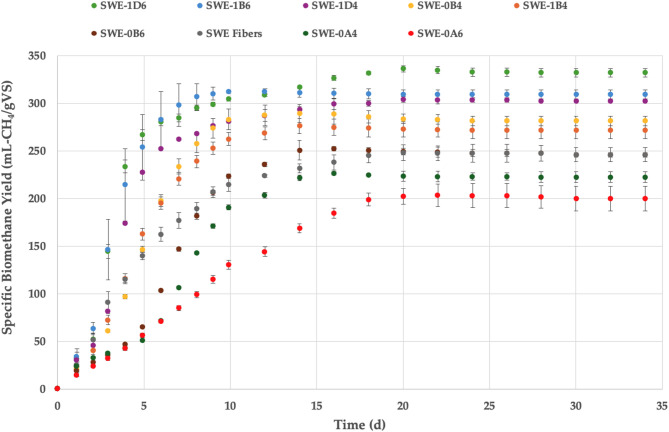
Specific biomethane yield in AD batch experiment of *S. ramosissima* fibers after SWE alone or SWE and subsequent organosolv fractionation. SLE, Soxhlet-extracted fibers; SWE, subcritical water-extracted fibers; 0, 200 °C; 1, 180 °C; A, 15 min; B, 30 min; D, 60 min; 4, 40% v/v ethanol; 6, 60% v/v ethanol.

Figure 8 illustrates the biomethane yields of lignified *S. ramosissima* fibers after SLE only or SLE and organosolv fractionation under eight different conditions. Likewise, Fig. 9 displays the biomethane yields of lignified *S. ramosissima* fibers that underwent SWE alone or SWE and organosolv pretreatments. The BMP of organosolv-pretreated lignified biomass was generally higher than the BMP of lignified fibers after extraction only.

Batch assay results indicated a faster degradation rate for most organosolv-treated lignified *S. ramosissima* fibers, showing a primary digestion of 13–15 days compared to 20–24 days for the fibers after extraction only (Figs. 8 and 9). This implies an improvement in both the rate and extent of hydrolysis during anaerobic breakdown of the residual fibers upon organosolv pretreatment. In SLE-0B4 (200 °C, 30 min, 40% v/v ethanol) and SLE-0B6 (200 °C, 30 min, 60% v/v ethanol) fibers, a lag phase lasting 4–6 days was noted, indicating that the microorganisms needed additional time to adapt and start hydrolysis of the polymers. The highest biomethane yield for SLE pulp was 302 mL-CH_4_/gVS for organosolv treatment at 180 °C for 60 min with 60% v/v ethanol (SLE-1D6), followed by 280 mL-CH_4_/gVS at 200 °C for 15 min with 60% v/v ethanol (SLE-0A6). In contrast, the methane yield of SLE pulp without subsequent organosolv was 174 mL-CH_4_/gVS, underscoring the importance of organosolv fractionation for increasing the methane yield by 28%–74%.

For residual fibers after SWE and organosolv pretreatment, the course of the batch assay exhibited no distinct lag phase (Fig. 9). In general, the methane yield from residual fibers was higher for SWE pulp than for SLE pulp. This may be attributed to the higher hemicellulose content in SWE fibers than in SLE fibers after organosolv and, consequently, their availability for microbial degradation. The methane yield of SWE fibers was highest when organosolv was performed at 180 °C for 60 min with 60% v/v ethanol (SWE-1D6; 336 mL-CH_4_/gVS), followed by 180 °C for 30 min with 60% v/v ethanol (SWE-1B6; 312 mL-CH_4_/gVS), and 180 °C for 60 min with 40% v/v ethanol (SWE-1D4; 304 mL-CH_4_/gVS). Subsequent organosolv fractionation of extractive-free fibers increased methane yields from 17% to 35% compared to 248 mL-CH_4_/gVS achieved with SWE pulp.

Pretreatment conditions play a vital role in improving the degradability of lignocellulosic biomass but can lead to the loss of organic material and the production of inhibitory compounds. Optimum methane yields were obtained from pretreated fibers at 180 °C for 60 min using 60% v/v ethanol (1D6) for both SWE and SLE. In contrast, the lowest methane yields were attained following organosolv at 200 °C for 30 min with 40% v/v ethanol for SLE-0B4 (170 mL-CH_4_/gVS) and at 200 °C for 15 min with 60% v/v ethanol for SWE-0A6 (202 mL-CH_4_/gVS). Increasing the residence time from 30 min (1B6) to 60 min (1D6) at a constant temperature (180 °C) with 60% v/v ethanol resulted in higher methane yields of SLE fibers. This is attributed to a higher cellulose content in pretreated fibers (1D6) and comparable degree of delignification. Similarly, SWE fibers led to enhanced methane yields as residence time increased from 30 min (1B6) to 60 min (1D6). At a lower solvent concentration of 40% v/v ethanol (1D4), both SLE and SWE fibers resulted in lower methane yields. This corresponded to a lower extent of delignification compared to using a higher solvent concentration (60% v/v ethanol). Comparatively, increasing the pretreatment temperature from 180 °C (1B6) to 200 °C (0B6) at a constant reaction time (30 min) and 60% v/v ethanol resulted in lower methane yields of SLE and SWE fibers. A lower temperature of 180 °C, along with a prolonged pretreatment duration of 60 min and a higher solvent concentration of 60% v/v ethanol, seems to establish optimal conditions for improving methane yields. Methane yields obtained from organosolv-treated fibers are typically associated with the effectiveness of fractionation. Similar to the findings by Cayenne et al.^19^, no correlation was found between methane yields and lignin content in pretreated lignified *S. ramosissima* fibers. Thus, the differences in BMP yields from pretreated fibers arise from the breaking of bonds among cellulose, hemicellulose, and lignin rather than from the specific lignin content.

Table 6 lists the theoretical and experimental methane yields, the percentage change in BMP, and the anaerobic biodegradability of lignified *S. ramosissima* fibers before and after different extraction processes and organosolv treatment. Generally, extraction followed by organosolv fractionation of lignified fibers led to enhanced methane yields. For SLE organosolv-treated fibers, methane yields increased by 28%–74%, while for SWE organosolv-treated fibers, yields increased by 2%–35%. This demonstrates the effectiveness of extraction plus organosolv fractionation in improving accessibility of cellulose and hemicellulose in pretreated fibers. SWE organosolv-treated fibers achieved a higher biodegradability (66%–127%) compared to SLE organosolv-treated fibers (65%–105%). The highest methane yield was achieved upon pretreatment at 180 °C for 60 min and 60% ethanol (1D6) for both SLE and SWE fibers. Therefore, this organosolv condition can be considered the most suitable to achieve high methane production from lignified fibers subjected to extraction of phytochemicals. In a previous study, the most suitable organosolv pretreatment conditions for de-juiced green fibers of *S. dolichostachya* and *S. ramosissima* were found to be 200 °C for 30 min or 180 °C for 60 min at 60% v/v ethanol, and 180 °C for 30 min with 60% v/v ethanol, which achieved 300 mL-CH_4_/gVS^19^. In the present study, extracted organosolv-treated lignified *S. ramosissima* fibers contained a higher cellulose content than pretreated de-juiced *S. ramosissima* green fibers^19^. This may indicate that organosolv fractionation is more effective for biomass with a higher lignin content.

**Table 6:** Theoretical and experimental methane yields, percentage change in BMP, and anaerobic biodegradability of lignified *S. ramosissima* fibers before and after different extraction processes and organosolv fractionations.

Halophytic Substrates	BMP_EXP_	ΔCH_4_	TBMP_OFC_	Biodegradability
	mL-CH_4_/gVS	%	mL-CH_4_/gVS	%
SLE Fibers	173.9 (6.2)	-	243	72
SLE-0A4	253.6 (1.6)	+ 46	248	102
SLE-0A6	280.1 (6.7)	+ 61	278	101
SLE-0B4	170.2 (7.5)	-2	261	65
SLE-0B6	222.7 (9.0)	+ 28	295	75
SLE-1B4	248.7 (1.8)	+ 43	288	86
SLE-1B6	266.7 (8.7)	+ 53	254	105
SLE-1D4	262.1 (11.4)	+ 51	272	96
SLE-1D6	302 (2.1)	+ 74	299	101
SWE Fibers	248.2 (1.2)	-	242	102
SWE-0A4	226.3 (4.9)	-8.8	220	103
SWE-0A6	202.1 (8.4)	-18.6	308	66
SWE-0B4	289.3 (9.4)	+ 16.6	227	127
SWE-0B6	252.1 (10.1)	+ 1.6	299	84
SWE-1B4	276.0 (6.8)	+ 11.2	266	104
SWE-1B6	312.1 (9.7)	+ 25.7	314	99
SWE-1D4	304.1 (3.1)	+ 22.5	265	115
SWE-1D6	336.1 (3.3)	+ 35.4	286	118

BMP_EXP_: experimental biomethane potential; TBMP_OFC_: theoretical biomethane potential; ΔCH_4_: % change in BMP. SLE, Soxhlet-extracted fibers; SWE, subcritical water-extracted fibers; 0, 200 °C; 1, 180 °C; A, 15 min; B, 30 min; D, 60 min; 4, 40% v/v ethanol; 6, 60% v/v ethanol.

## Conclusion

Process integration in a biorefinery framework offers a promising approach to enhance efficiency and sustainability of marketable products. In this study, we evaluated the combined effect of different pretreatments for extractives removal (i.e., SLE and SWE), followed by organosolv fractionation under different processing parameters. We obtained higher fractionation of hemicelluloses with SLE fibers. Both extractions created conditions that allowed for low cellulose removal (< 6% SLE-1D6, SWE-1B4, SWE-1B6, SWE-1D4), but high (> 69%) delignification (SLE-0A6, SLE-0B6, SLE-1B6, SLE-1D6 and SWE-0A6). Subsequently, we evaluated the generated pulp in terms of enzymatic saccharification and, hence, suitability as feedstock for renewable energy production via microbial anaerobic digestion. We obtained methane yields > 300 mL-CH_4_/gVS for both extraction methods, with highest values for SWE samples. SWE samples achieved also full saccharification in enzymatic trials. Overall, this study demonstrates the successful processing of *S. ramosissima*, a biomass not previously explored in a biorefinery concept, as having high potential in the synergistic integration of different technologies to generate building blocks for value-added products. This example will facilitate the transition to a circular economy.

## Data Availability

The data supporting the findings of this study are available within the article.

## References

[1] Werpy, T. & Petersen, G. *Top Value Added Chemicals from Biomass: Volume I—Results of Screening for Potential Candidates from Sugars and Synthesis Gas*. 10.2172/15008859 (2004).

[2] Butnariu, M. & Butu, A. Chemical composition of vegetables and their products. In *Handbook of Food Chemistry* 1–49 (Springer, 2014). 10.1007/978-3-642-41609-5_17-1.

[3] Hounsome, N., Hounsome, B. & Lobo, M. G. Biochemistry of vegetables. In *Handbook of Vegetables and Vegetable Processing* 25–46 (Wiley, 2018). 10.1002/9781119098935.ch2.

[4] Kalt, W. Effects of production and processing factors on major fruit and vegetable antioxidants. *J. Food Sci.* **70**, R11–R19 (2005).

[5] Phillips, K. M. et al. Seasonal variability of the vitamin C content of fresh fruits and vegetables in a local retail market. *J. Sci. Food Agric.* **98**, 4191–4204 (2018). 29406576 10.1002/jsfa.8941

[6] Palacio, S. et al. Seasonal variability of dry matter content and its relationship with shoot growth and nonstructural carbohydrates. *New Phytol.* **180**, 133–142 (2008). 18643937 10.1111/j.1469-8137.2008.02569.x

[7] Zhang, Y. H. P. Reviving the carbohydrate economy via multi-product lignocellulose biorefineries. *J. Ind. Microbiol. Biotechnol.* **35**, 367–375 (2008). 18180967 10.1007/s10295-007-0293-6

[8] Stødkilde, L., Lashkari, S., Eriksen, J. & Jensen, S. K. Enhancing protein recovery in green biorefineries through selection of plant species and time of harvest. *Anim. Feed Sci. Technol.* **278**, 115016 (2021).

[9] Marvin, W. A., Schmidt, L. D. & Daoutidis, P. Biorefinery Location and technology selection through supply chain optimization. *Ind. Eng. Chem. Res.* **52**, 3192–3208 (2013).

[10] Hulkko, L. S. S. et al. Cultivation and characterisation of *Salicornia europaea*, *Tripolium pannonicum* and *Crithmum maritimum* biomass for green biorefinery applications. *Sci. Rep.* **12**, 20507 (2022). 36443447 10.1038/s41598-022-24865-4 PMC9705282

[11] Hulkko, L. S. S. et al. Bioactive extracts from *Salicornia ramosissima* J. woods biorefinery as a source of ingredients for high-value industries. *Plants* **12**, 1251 (2023). 36986939 10.3390/plants12061251 PMC10056203

[12] Giordano, R. et al. Effects of salicornia-based skin cream application on healthy humans’ experimental model of pain and itching. *Pharmaceuticals* **15**, 150 (2022). 35215262 10.3390/ph15020150 PMC8876271

[13] Ishikawa, N., Shimizu, K., Koizumi, T., Shimizu, T. & Enishi, O. Nutrient value of saltwort (*Salicornia herbacea* L.) as feed for ruminants. *Asian Australas J. Anim. Sci.* **15**, 998–1001 (2002).

[14] Barreto, A. et al. *Salicornia ramosissima* biomass as a partial replacement of wheat meal in diets for juvenile European seabass (*Dicentrarchus labrax*). *Animals* **14**, 614 (2024). 38396582 10.3390/ani14040614 PMC10886228

[15] Kim, Y. A. et al. Evaluation of *Salicornia herbacea* as a potential antioxidant and anti-inflammatory agent. *J. Med. Food* **12**, 661–668 (2009). 19627218 10.1089/jmf.2008.1072

[16] Singh, S. et al. Biodiversity and antimicrobial potential of bacterial endophytes from halophyte *Salicornia brachiata*. *Antonie Van Leeuwenhoek*. **114**, 591–608 (2021). 33674993 10.1007/s10482-021-01544-4

[17] Monção, M., Wretborn, T., Rova, U., Matsakas, L. & Christakopoulos, P. Salicornia dolichostachya organosolv fractionation: Towards establishing a halophyte biorefinery. *RSC Adv.* **12**, 28599–28607 (2022). 36320546 10.1039/d2ra04432c PMC9540244

[18] Monção, M. et al. A novel biorefinery concept based on marginally used halophyte biomass. *Sustain. Energy Fuels* **7**, 3902–3918 (2023).

[19] Cayenne, A. et al. Enhancing the methane yield of *Salicornia* spp. via organosolv fractionation as part of a halophyte biorefinery concept. *Energies* **17**, 1074 (2024).

[20] Turcios, A. E., Cayenne, A., Uellendahl, H. & Papenbrock, J. Halophyte plants and their residues as feedstock for biogas production-chances and challenges. 10.3390/app11062746 (2021).

[21] Cayenne, A. et al. Halophytes as feedstock for biogas production: Composition analysis and biomethane potential of *Salicornia* spp. plant material from hydroponic and seawater irrigation systems. *Fermentation* **8**, 189 (2022).

[22] Mehta, D. & Vyas, S. Comparative bio-accumulation of osmoprotectants in saline stress tolerating plants: A review. *Plant Stress* **9**, 100177 (2023).

[23] Turcios, A. E. et al. Compositional changes in hydroponically cultivated *Salicornia europaea* at different growth stages. *Plants* **12**, 2472 (2023). 37447033 10.3390/plants12132472 PMC10346760

[24] Fredsgaard, M., Kaniki, S. E. K., Antonopoulou, I., Chaturvedi, T. & Thomsen, M. H. Phenolic compounds in *Salicornia* spp. and their potential therapeutic effects on H1N1, HBV, HCV, and HIV: A review. *Molecules* **28**, 5312 (2023). 37513186 10.3390/molecules28145312 PMC10384198

[25] Choi, D. et al. Characterization, stability, and antioxidant activity of *Salicornia herbaciea* seed oil. *Korean J. Chem. Eng.* **31**, 2221–2228 (2014).

[26] AlYammahi, J. et al. Salicornia seed oil: A high-yielding and sustainable halophytic feedstock for biodiesel and energy in underutilized hypersaline coastal deserts. *Energy Convers. Manag.* **318**, 118914 (2024).

[27] Singh, D., Buhmann, A. K., Flowers, T. J., Seal, C. E. & Papenbrock, J. Salicornia as a crop plant in temperate regions: Selection of genetically characterized ecotypes and optimization of their cultivation conditions. *AoB Plants* **6**, plu071 (2014). 10.1093/aobpla/plu071 PMC4268490 25387752

[28] Ventura, Y. & Sagi, M. Halophyte crop cultivation: The case for *Salicornia* and *Sarcocornia*. *Environ. Exp. Bot.* **92**, 144–153 (2013).

[29] Marques, B., Maciel, E., Domingues, M. R., Calado, R. & Lillebø, A. I. Halophyte plants cultured in aquaponics hold the same potential for valorization as wild conspecifics from donor sites. *Appl. Sci.* **11**, 11586 (2021).

[30] Puccinelli, M. et al. Growing *Salicornia europaea* L. with saline hydroponic or aquaculture wastewater. *Horticulturae* **10**, 196 (2024).

[31] Ventura, Y. et al. Effects of day length on flowering and yield production of *Salicornia* and *Sarcocornia* species. *Sci. Hortic.* **130**, 510–516 (2011).

[32] Azwanida, N. N. A Review on the extraction methods use in medicinal plants, principle, strength and limitation. *Med Aromat. Plants* **4**, 2167 (2015).

[33] Hawthorne, S. B., Grabanski, C. B., Martin, E. & Miller, D. J. Comparisons of Soxhlet extraction, pressurized liquid extraction, supercritical fluid extraction and subcritical water extraction for environmental solids: Recovery, selectivity and effects on sample matrix. *J. Chromatogr. A*. **892**, 421–433 (2000). 11045502 10.1016/s0021-9673(00)00091-1

[34] Fredsgaard, M., Tchoumtchoua, J., Kohnen, S., Chaturvedi, T. & Thomsen, M. H. Isolation of polyphenols from aqueous extract of the halophyte *Salicornia ramosissima*. *Molecules* **29**, 220 (2023). 38202803 10.3390/molecules29010220 PMC10780970

[35] Nair, L. G., Agrawal, K. & Verma, P. Organosolv pretreatment: An in-depth purview of mechanics of the system. *Bioresour. Bioprocess.* **10**, 50 (2023). 38647988 10.1186/s40643-023-00673-0 PMC10991910

[36] Tofani, G., Jasiukaitytė-Grojzdek, E., Grilc, M. & Likozar, B. Organosolv biorefinery: Resource-based process optimisation, pilot technology scale-up and economics. *Green Chem.* **26**, 186–201 (2024).

[37] Paulsen Thoresen, P. et al. Characterization of organosolv birch lignins: Toward application-specific lignin production. *ACS Omega* **6**, 4374–4385 (2021). 33623848 10.1021/acsomega.0c05719 PMC7893791

[38] Jasiukaitytė-Grojzdek, E. et al. Design of organosolv lignin fractionation: Influence of temperature, antisolvent, and source on molecular weight, structure, and functionality of lignin fragments. *ACS Sustain. Chem. Eng.* **13**, 3452–3466 (2025).

[39] Abbas, A., Wang, Z., Zhang, Y., Peng, P. & She, D. Lignin-based controlled release fertilizers: A review. *Int. J. Biol. Macromol.* **222**, 1801–1817 (2022). 36191787 10.1016/j.ijbiomac.2022.09.265

[40] Pazzaglia, A. et al. Wood waste valorization: Ethanol based organosolv as a promising recycling process. *Waste Manag.* **170**, 75–81 (2023). 10.1016/j.wasman.2023.08.003 37552928

[41] Islam, M., Sinha, A. S. K. & Prasad, K. Organosolv delignification of rice straw cellulose fiber for functional food packaging. *Cellulose* **31**, 9191–9214 (2024).

[42] Weinwurm, F. et al. Lignin concentration and fractionation from ethanol organosolv liquors by ultra- and nanofiltration. *J. Clean. Prod.* **136**, 62–71 (2016).

[43] Akgul, M. & Kirci, H. An environmentally friendly organosolv (ethanol-water) pulping of poplar wood. *J. Environ. Biol.* **30**, 735–740 (2009). 20136058

[44] Sluiter, A. et al. Determination of structural carbohydrates and lignin in biomass: Laboratory analytical procedure (LAP). http://www.nrel.gov/biomass/analytical_procedures.html (2008).

[45] Sluiter, A. et al. Determination of sugars, byproducts, and degradation products in liquid fraction process samples (2006).

[46] Sluiter, A., Ruiz, R., Scarlata, C., Sluiter, J. & Templeton, D. *Determination of Extractives in Biomass* (2005).

[47] Deutsches Institut für Normung. *Characterization of Sludges - Determination of the Loss on Ignition of Dry Mass* Vol. 12879 (Deutsches Institut für Normung, 2000).

[48] Deutsches Institut für Normung. *Characterization of Sludges - Determination of Dry Residue and Water Content* Vol. 12880 (Deutsches Institut für Normung, 2001).

[49] Meng, X. et al. Physicochemical structural changes of poplar and switchgrass during biomass pretreatment and enzymatic hydrolysis. *ACS Sustain. Chem. Eng.* **4**, 4563–4572 (2016).

[50] Deutsches Institut für Normung. *Fermentation of Organic Materials – Characterization of the Substrate, Sampling, Collection of Material Data, Fermentation Tests* Vol. 4630 (Deutsches Institut für Normung, 2016).

[51] Lesteur, M. et al. Alternative methods for determining anaerobic biodegradability: A review. *Process Biochem.* **45**, 431–440 (2010).

[52] Raposo, F. et al. Biochemical methane potential (BMP) of solid organic substrates: Evaluation of anaerobic biodegradability using data from an international interlaboratory study. *J. Chem. Technol. Biotechnol.* **86**, 1088–1098 (2011).

[53] Muniz Kubota, A., Kalnins, R. & Overton, T. W. A biorefinery approach for fractionation of *Miscanthus* lignocellulose using subcritical water extraction and a modified organosolv process. *Biomass Bioenergy* **111**, 52–59 (2018).

[54] Duarte, L. C., Sampaio, B. & Carvalheiro, F. Organosolv pretreatment of lignocellulosic biomass. In H*andbook of Biorefinery Research and Technology: Biomass Logistics to Saccharification* 487–514 (Springer, 2024) 10.1007/978-94-007-6308-1_81 (2024).

[55] Rasmussen, H., Sørensen, H. R. & Meyer, A. S. Formation of degradation compounds from lignocellulosic biomass in the biorefinery: Sugar reaction mechanisms. *Carbohydr. Res.* **385**, 45–57 (2014). 24412507 10.1016/j.carres.2013.08.029

[56] Tchuidjang, T. T., Noubissié, E. & Ali, A. Optimization of the pre-treatment of white sawdust (*Triplochiton scleroxylon*) by the organosolv process for the production of bioethanol. *Oil Gas Sci. Technol. Revue d’IFP Energies Nouvelles* **76**, 23 (2021).

[57] Chen, Y., Cheng, J. J. & Creamer, K. S. Inhibition of anaerobic digestion process: A review. *Bioresour. Technol.* **99**, 4044–4064 (2008). 17399981 10.1016/j.biortech.2007.01.057

